# Enhanced microvascular imaging through deep learning-driven OCTA reconstruction with squeeze-and-excitation block integration

**DOI:** 10.1364/BOE.525928

**Published:** 2024-09-03

**Authors:** Mohammad Rashidi, Georgy Kalenkov, Daniel J. Green, Robert A. McLaughlin

**Affiliations:** 1 Faculty of Health and Medical Sciences, The University of Adelaide, Adelaide SA 5005, Australia; 2 Institute for Photonics and Advanced Sensing, The University of Adelaide, Adelaide SA 5005, Australia; 3 School of Human Sciences (Exercise and Sport Sciences), The University of Western Australia, Crawley WA 6009, Australia; 4 School of Engineering, The University of Western Australia, Crawley WA 6009, Australia

## Abstract

Skin microvasculature is essential for cardiovascular health and thermoregulation in humans, yet its imaging and analysis pose significant challenges. Established methods, such as speckle decorrelation applied to optical coherence tomography (OCT) B-scans for OCT-angiography (OCTA), often require a high number of B-scans, leading to long acquisition times that are prone to motion artifacts. In our study, we propose a novel approach integrating a deep learning algorithm within our OCTA processing. By integrating a convolutional neural network with a squeeze-and-excitation block, we address these challenges in microvascular imaging. Our method enhances accuracy and reduces measurement time by efficiently utilizing local information. The Squeeze-and-Excitation block further improves stability and accuracy by dynamically recalibrating features, highlighting the advantages of deep learning in this domain.

## 1. Introduction

The optimal function and health of the skin depend on cutaneous microvasculature [1]. Perturbations or abnormalities in these small blood vessels can significantly impact a range of dermatologic conditions [2], including skin lesions [3] and psoriasis [4]. Additionally, various systemic diseases, such as diabetes [5] and Raynaud's phenomenon [6], can also contribute to alterations or irregularities in these small blood vessels. Imaging the microvasculature is important because it can enable early detection and precise assessment of microvascular dysfunction, facilitating early diagnosis to optimize and personalize treatment strategies for dermatologic and systemic conditions. Optical coherence tomography angiography (OCTA) is a powerful imaging modality [7–9] in this context. It is non-invasive, and its micron-scale resolution capabilities provide clinicians and researchers with visualization of the cutaneous microvasculature, allowing for detailed analysis of blood flow patterns and vessel morphology [10]. This level of precision is instrumental in identifying subtle microvascular abnormalities, guiding treatment decisions, and monitoring therapeutic responses in both dermatologic and systemic diseases.

OCTA utilizing speckle intensity relies on contrasting temporal changes in the backscattered light properties of static and dynamic regions to visualize blood flow. In imaging systems such as optical coherence tomography (OCT) [11], speckles emerge from the interference of light waves backscattered by random scatterers, resulting in variations in light intensity or brightness [12]. Studies have indicated that temporal variations in speckle patterns contain information about the movement of scattering particles [13,14]. Specifically, the speckle pattern remains relatively consistent over time for stationary objects, whereas it changes when objects are in motion. Various techniques, such as calculating the variance [15] or correlation [16] of speckle intensity over a temporal window, have been employed to quantify these changes. For example, Mariampillai *et al.* [15] calculated speckle variance over three consecutive B-scans (N = 3) to differentiate blood vessels (high speckle variance) from surrounding static tissues (low speckle variance). In a different study, Enfield *et al.* [16] used correlation mapping of two consecutive B-scans (N = 2) to extract capillary density and vessel diameter in the *in vivo* human volar forearm. Not only can speckle changes qualitatively help in differentiating vessels from static regions, but they can also be used to quantify blood flow, although these applications can be challenged by the issues of low signal-to-noise ratio (SNR) and noise in OCTA data [8,17]. Specifically, noise can affect the speckle statistics and introduce random variations in the intensity values of OCTA images, compromising the accuracy of vascular analysis and the detection of subtle vascular features. For example, it has been shown that the SNR in the processed OCTA images obtained from speckle analysis relies on the number of acquisitions (N) at the same positions [18]. Increasing N has been shown to improve image SNR by a factor of $\sqrt{N}$ , making it important for capturing high-contrast images of small vessels. However, using a high N value can be time-consuming and susceptible to motion artifacts caused by involuntary patient movements, leading to a trade-off between motion-induced noise and the potential increase in noise due to the limited amount of acquired information (low N value) in this method.

In traditional speckle variance and decorrelation calculations, estimates of the speckle decorrelation at each location are performed on data within a sliding window. The choice of window size tends to be application-specific, as a larger window provides more data for better estimation of the speckle statistics at each location, but reduces the spatial resolution of the estimate and suffers reduced accuracy due to partial volume effects. An alternative approach to using a fixed-size window is to exploit deep learning methods to use local information more effectively. This strategy enhances vessel detection accuracy by capitalizing on spatial relationships between pixels, potentially surpassing methods heavily reliant on the sliding filter, which may miss valuable local context. Beyond this, deep learning brings additional advantages, such as automatic feature learning, and adaptability to diverse data. These attributes make deep learning a promising approach for reducing the required number of B-scans in OCTA, optimizing efficiency while maintaining or enhancing reconstruction accuracy. In a study conducted by Liu *et al.* [19], a deep learning framework was proposed for OCTA reconstruction, utilizing sequential B-scans. The findings highlighted the superior performance of the deep learning approach compared to traditional speckle decorrelation methods. Notably, the study revealed that utilizing only N = 2 B-scans with deep learning yielded reconstruction results surpassing those obtained with N = 4 B-scans using speckle decorrelation techniques. Moreover, deep learning models can not only enhance the quality of reconstructed images with fewer B-scans but also improve resolution, generating high-resolution angiograms from undersampled data [20,21]. This emphasizes the effectiveness of deep learning methodologies in enhancing both the quality and resolution of OCTA reconstruction processes.

While previous studies have demonstrated the potential of deep learning-based models to enhance processing speed and vessel contrast in OCTA [22–25], these models were restricted to using conventional architectures that emphasize spatial correlations and multi-scale processes but may neglect interdependencies between channels. In the terminology of deep learning, a ‘channel’ denotes a specific dimension of the input data. For example, in a standard video image, each channel may correspond to a distinct color (e.g., red, green, blue). In our application, each co-located B-scan corresponds to a distinct channel. Previous studies have shown that incorporating a Squeeze-and-Excitation (SE) block into deep learning models effectively mitigates the limitation of neglecting inter-channel dependencies in traditional architectures [26]. The SE block facilitates dynamic channel-wise feature recalibration, which enables the model to prioritize crucial input elements through an attention mechanism and regulate information flow within the network via a gating mechanism [26]. This adaptive process enables the network to assign varying degrees of importance to different channels based on their relevance. As a result, it notably improves the network's capacity to capture essential features and spatial correlations. The SE block has been demonstrated as an effective way to learn the relationship between channels in other imaging modalities (i.e video [27], MRI [28], CT [29]). However, unlike many imaging modalities, OCT is characterized by its speckle phenomena. This has inherently different noise statistics, as the signal from a single tissue type will vary across the full range of potential values as the light waves constructively and destructively interfere. Previous work has not established whether the SE block is able to characterize inter-channel relationships given such a signal. Note that other approaches are possible to capture the relationship between channels, and hence co-located B-scans, such as self-attention and transformer architectures [30]. These have the advantage of more naturally capturing long-range relationships [31], at a cost of greater training requirements. However, the small blood vessels of the microvasculature are inherently highly spatially localized in nature, making the SE block an appropriate choice for exploring the value of learning inter-channel relationships.

In our study, we build upon insights from previous work [26] by implementing a Convolutional Neural Network (CNN) integrated with the SE block for OCTA reconstruction. We train the neural network on the best estimate of the true value of speckle decorrelation (using all available data), and then explore the capability of the network to predict this value given progressively reduced amounts of OCT input data. Our findings demonstrate that employing neural networks (NNs) instead of traditional speckle decorrelation algorithms notably enhances accuracy, especially in scenarios with a low number of channels. Aligned with our primary goal of reducing acquisition time through fewer B-scans, our results affirm the effectiveness of NN in this context. The primary contribution of our study lies in illustrating that the integration of the SE block not only improves stability and reduces overfitting but also could improve accuracy compared to the conventional CNN without an SE block. This novel application of the SE block in OCTA reconstruction increases the scope of NN architectures available for OCT applications.

## 2. Data preparation

### 2.1 OCT hardware and imaging setup

**Fig. 1.**
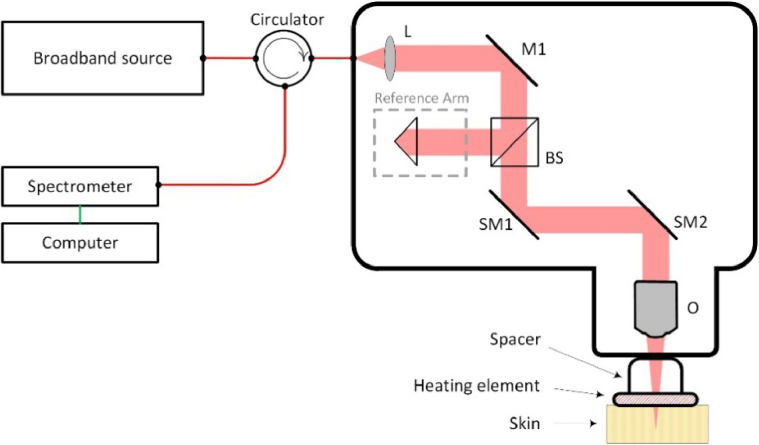
Schematic representation of the spectral domain optical coherence tomography system. Internal details of the scanhead are enclosed within the thick black outline. A bespoke spacer with an embedded heating element is positioned directly on the skin to facilitate localized heating for vasodilation studies. The setup comprises a source, a spectrometer connected to a computer, a lens - (L), a mirror - (M1), a beam splitter - (BS), scanning mirrors - (SM1 and SM2), and an objective - (O).

Figure 1 illustrates the hardware configuration and imaging setup used for data acquisition. We utilized a commercial spectral domain optical coherence tomography system (Telesto TEL321C1, Thorlabs GmbH, Germany), with a central wavelength of 1300 nm and an intrinsic axial resolution of 5 µm in tissue (assuming a refractive index of 1.43). The optical configuration of the core OCT system had not been modified. The scan head provides a lateral resolution of 13 µm and a focal length of 36 mm. The OCT system comprises a broadband source connected to a circulator. The circulator feeds the optical signal into a scanhead that includes a lens (L), a reference arm, a beam splitter (BS), scanning mirrors (SM1 and SM2), and an objective (O) directed towards the sample (skin). The reflected light from the sample and the reference arm are recombined at the beam splitter and directed into the spectrometer, and the digitized signal is reconstructed to generate an OCT C-scan. To maintain a consistent position relative to the skin, a custom spacer with an integrated heating element is used. This element can warm the skin to 42 degrees Celsius, which has been previously shown to induce vasodilation and increase blood flow [32]. We acquired two types of scans: baseline scans acquired at room temperature; and heated scans after the skin had been locally heated and vasodilated for 30 minutes, showing increased rates of blood flow.

### 2.2 OCTA dataset and experimental protocol

Five healthy participants (one female and four males, age range 21-63yo), were included in the study. This study was approved by The University of Western Australia’s Human Research Ethics Committee and conformed to the standards outlined in the Declaration of Helsinki. The participants lay supine for at least 10 minutes with their arm supported by a foam pad, with the intention of minimizing movement during scanning. The OCT scanhead was placed on the area of interest on the volar forearm, distal to the elbow, and angled to lie flush with the subject’s skin. As shown in Fig. 1, a spacer and small heating element held the scanhead at a fixed distance from the skin surface. A few drops of water were used between the skin and the glass window of the spacer to reduce any refractive index mismatches.

**Data Acquisition:** 3D OCT C-scans were acquired with one of two field-of-view settings, depending on the scanning setup. These were either a 5 mm × 5 mm × 2.5 mm (length × width × depth) field of view or a field of view of 3.2 mm × 3.2 mm × 2.5 mm. Each B-scan was discretized as 1000 × 1024 (x × z) pixels. A block of 10 B-scans were acquired at each y location (slow axis), with a spacing of 10 µm between B-scan blocks.

**Fig. 2.**
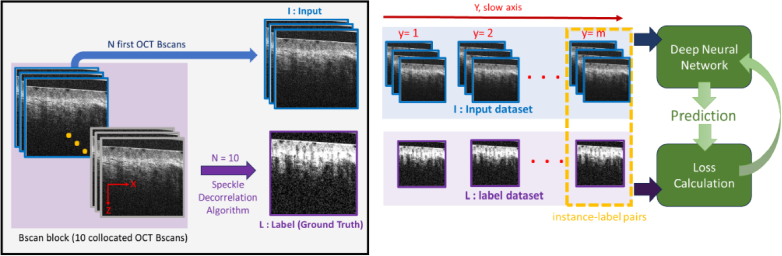
Schematic illustration of the OCTA pipeline employing deep learning: (left) preparing the instance-label pairs from the B-scan block. (right) Training the neural network with the prepared instance-label pairs.

**Data Preprocessing:** B-scan blocks were separated into distinct Training, Validation and Testing sets. For Training data, all 10 B-scans in each B-scan block were used to estimate the speckle decorrelation at each (x × z) location. Specifically, the L1-norm (absolute difference in OCT intensity) was calculated between pairs of sequentially acquired B-scans within a block. These values were averaged over all pairs to provide the best estimate of speckle decorrelation. For Validation and Testing data, different numbers of B-scan pairs within each block were used, allowing assessment of prediction error with increasing amounts of OCT data. Furthermore, the OCT intensity data underwent normalization, ensuring values ranged between 0 and 1 before input into the model. The training and validation datasets were designed to include both baseline (B) and heated (H) samples. Data from an acquisition were kept exclusive to either the training, validation, or test datasets. As shown in Table 1, our training dataset included 9 datasets, and the validation set had 2 datasets. This arrangement resulted in a total of 3780 cross-sectional image pairs for training and 820 for validation. The test dataset comprised 2 distinct data acquisitions: one heated sample with 500 cross-sectional image pairs and one baseline sample with 320 cross-sectional image pairs. Each pair consists of input OCT B-scan blocks, comprising N consecutive B-scans at each slow axis location, along with their corresponding label image represented by speckle decorrelation maps derived from 10 B-scans (Fig. 2).

**Table 1.** The training, validation, and test datasets’ information

Participant	Gender	Number of Samples			Length (width) size, mm
		Training	Validation	test	
Subject 1	M	3 H, 1 B	1 B	1 B	3.2
Subject 2	F	1 H	1 H		5
Subject 3	M	1 B	-		3.2
Subject 4	M	2 H, 1 B	-		5
Subject 5	M	-	-	1 H	5

All the networks were implemented via Pytorch (https://pytorch.org/) on NVIDIA GPUs (GeForce RTX(TM) 3080 Ti, https://www.nvidia.com/).

## 3. Methods

### 3.1 OCTA reconstruction model

To obtain speckle decorrelation values for the analysis of OCTA data, two distinct methods were employed: a conventional speckle decorrelation algorithm and a deep learning-based approach utilizing different neural network architectures.

#### 3.1.1 Conventional speckle decorrelation algorithm

The conventional speckle decorrelation algorithm involved the use of a sliding window technique. A 6 × 6 (x × z) filter was swept across each B-scan block, consisting of N B-scans, and decorrelation values were calculated by measuring changes in speckle patterns among these N B-scans within the defined window. By varying N within the range of 2 to 10, we were able to explore the impact of the number of measurements on speckle decorrelation accuracy. The speckle decorrelation calculation used the L1-norm, calculating the average of the absolute values of intensity differences within the 6 × 6 window. This traditional method provides a baseline for comparison with the deep learning-based results, offering insights into the efficacy of the NN in capturing speckle decorrelation patterns.

#### 3.1.2 Deep learning-based pipeline

Here, we employed a pipeline based on deep-learning techniques to generate the OCTA images [19]. This pipeline treats OCTA reconstruction as an end-to-end image translation task, encompassing three key phases: training data preparation, model learning, and OCTA prediction.

### 3.2 Training data preparation and preprocessing

The learning set consisted of instance-label pairs ${\left\{(I^{m},L^{m})\right\}}_{m=1}^{M}$ , where each pair corresponds to a ground truth image $L\in R^{H\times W}$ and its associated multi-channel OCT structural image $I\in R^{N\times H\times W}$ . In this context, each channel denotes a B-scan in the OCT structural image, representing cross-sectional X-Z images acquired at specific locations over time. Here, *m* represents the index of the image pair, while *N*, *H,* and *W* denote the respective numbers of input channels, rows, and columns in the image.

To enhance the efficiency of the training process, we implemented preprocessing techniques on the input data. This involved generating two overlapped 2D patches sized at 512 × 512 in the X-Z plane from each input B-scan, with a specified overlap size of 160 in the X-direction. The pair of overlapped patches were treated as two distinct, non-collocated subsets of the data. The input to the neural network comprised a block of collocated patches generated across 10 B-scans. The X-Z plane and a one set of collocated patches used as input to the deep neural network are illustrated in Fig. 2. This approach was undertaken to improve the model's capacity to encompass extensive spatial information, thereby facilitating enhanced contextual information and robust feature acquisition. This patch extraction approach facilitates more effective utilization of available data, leading to enhanced model performance in tasks reliant on spatial relationships [33].

Cross-sectional image pairs were acquired at locations along the slow-axis (Y-axis), where a series of 10 consecutive B-scans were captured and registered. Subsequently, a speckle decorrelation algorithm was employed to produce high SNR label angiograms (indexed here by L). The initial N-registered B-scans were utilized as input multi-channel images (indexed here by I), as illustrated in Fig. 2, and were fed into the neural network for further analysis.

### 3.3 Model learning

**Fig. 3.**
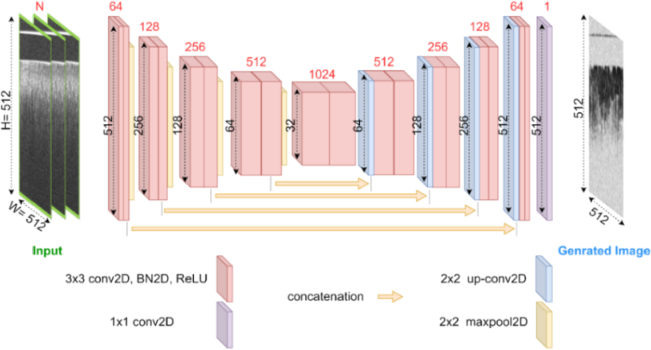
The architecture of the conventional 2D-Unet.

**Fig. 4.**
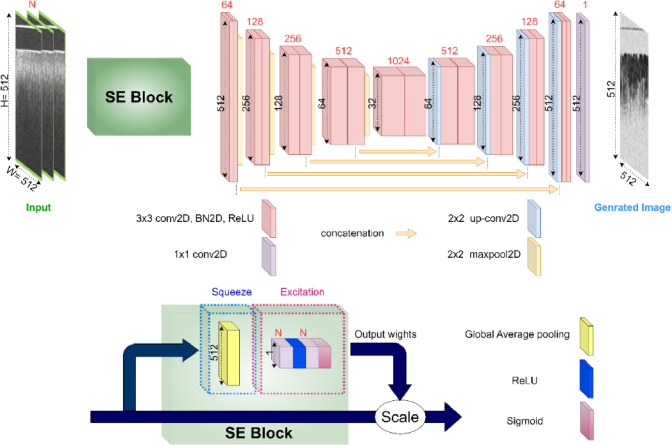
The architecture of the 2D-Unet with SE block.

The learning set was divided into training and validation sets. In our model implementation, we assessed two different encoder-decoder NNs: the conventional 2D-Unet (Fig. 3) and our proposed 2D-Unet with SE block (Fig. 4). The decision to incorporate the conventional 2D-Unet stemmed from its previous success in OCTA construction tasks, particularly in OCT to OCTA translation [22,23,25]. In comparative studies, the U-net demonstrated superior performance in terms of peak signal-to-noise ratio (PSNR) and structural similarity index measure (SSIM) compared to other deep learning architectures [23]. Additionally, the U-net architecture is well-suited for the incorporation of SE blocks, making it a practical choice for further performance enhancement [34,35].

The integration of the SE block was motivated by its demonstrated ability to enhance image segmentation and classification tasks [26]. The SE block's key advantages lie in its explicit modeling of interdependencies between channels, providing improved feature recalibration. This mechanism enables the selective use of global information, emphasizing informative features while suppressing less useful ones. Unlike the conventional speckle decorrelation algorithm, which makes rudimentary use of local information, both the conventional 2D Unet and the SE block-based Unet utilize local information more effectively. The SE block-based Unet goes a step further by incorporating channel-wise information as well. By incorporating a channel-wise weighting strategy, the SE block-based Unet enhances feature representation, enabling the model to extract more nuanced and informative features from each channel. During training, we minimized the mean squared error (MSE) loss between the generated speckle decorrelation map and the label image. The training process involved forward propagation, loss function calculation, and backpropagation, with model performance monitored using the MSE on the validation set.

In the implementation of our model, we employed a batch size of 2 and conducted training for 20 epochs with a learning rate of 5e-4. The Adam optimizer with a weight decay of 1e-5 was employed to minimize the MSE loss between the generated speckle decorrelation map and the label image. The label image was calculated using the speckle decorrelation algorithm using all 10 co-located B-scans from each B-scan block (Fig. 2). Network training is performed to minimize this MSE loss, reflecting the dissimilarity between the generated and ground truth speckle decorrelation maps. Additionally, a learning rate scheduler was utilized to dynamically adjust the learning rate during training based on the monitored validation loss. If no improvement was observed for two consecutive epochs, the learning rate was reduced by a factor of 0.1. This adaptive learning rate strategy aimed to enhance convergence and mitigate the risk of overshooting the optimal parameters, contributing to the stability and efficiency of the optimization process.

### 3.4 OCTA prediction

In the final phase of our deep learning-based OCTA pipeline, the trained encoder-decoder NNs, specifically the 2D-Unet, or the 2D-Unet with SE block, were used to predict OCTA images. The prediction process involved feeding OCT structural images from an unseen test dataset (Table 1) through the decoder part of the networks, generating predicted OCTA images. It's essential to note that the test datasets were kept separate and unseen during the training phase, ensuring an unbiased assessment of the model's generalization capability.

Before evaluating the accuracy of the OCTA predictions using quantitative metrics such as the structural similarity index measure (SSIM) [36] and peak signal-to-noise ratio (PSNR) [37], several preprocessing steps were applied. Firstly, both the NN predictions and the ground truth labels were normalized and then scaled to the grayscale range (i.e., L = 255) to facilitate fair comparison across different NNs and input N values. To compute the PSNR, we utilized the MSE derived from these normalized grayscale images. This involved scaling the predictions generated by the NN and the ground truth labels to a range of [0, 255]. The PSNR formula is given by:


(1) $$PSNR=10\log_{10}(\frac{L^{2}}{MSE_{normalized}})$$

(2) $$MSE_{normalized}=\frac{1}{n}\sum {\left(\frac{O_{label}-\min(O_{label})}{\max(O_{label})-\min(O_{label})}\times 255-\frac{O_{prediction}-\min(O_{prediction})}{\max(O_{prediction})-\min(O_{prediction})}\times 255\right)}^{2}$$

where O_label_ and O_prediction_ denote the output label calculated using the speckle decorrelation algorithm and the output prediction generated by the neural network, respectively, at each slow axis location.

In the SSIM calculation, the parameters were empirically set as follows: k_1 _= 0.01 and k_2 _= 0.03 and since the images were grayscale, L = 255 was chosen. Additionally, a Gaussian window of size 11 × 11 was selected, and SSIM values were calculated within these patches. The SSIM values were then averaged over these patches for each pair of B-scan block input and label. The SSIM formula is specified as:


(3) $$SSIM(O_{label},O_{prediction})=\frac{\left(2\mu_{label}\mu_{prediction}+c_{1}\right)}{\left({\mu_{label}}^{2}+{\mu_{prediction}}^{2}+c_{1}\right)}\frac{\left(2\sigma_{label,prediction}+c_{2}\right)}{\left({\sigma_{label}}^{2}+{\sigma_{prediction}}^{2}+c_{2}\right)}$$

where µ_label_ and µ_prediction_ are the mean values of the label and NN prediction, respectively, $\sigma_{label}^{2}$ and $\sigma_{prediction}^{2}$ are the variance of the label and NN prediction, $\sigma_{label,prediction}$ is the covariance of the label and NN prediction, and c_1_ and c_2_ are constants to stabilize the division with weak denominator. The relationship between c_1_ and c_2_ and k_1_ and k_2_ is given by:


(4) $$c_{1}={\left(k_{1}.L\right)}^{2},\;c_{2}={\left(k_{2}.L\right)}^{2}$$

These metrics were used to quantitatively assess the accuracy of OCTA predictions. Subsequently, to focus the evaluation on the high signal tissue region, which is typically close to the skin surface, the images were cropped to include only the area from the surface of the skin up to ∼ 750 µm (150 pixels) below the skin. The SNR of the OCT data was found to degrade rapidly beyond this depth in the highly scattering skin due to multiple scattering.

## 4. Results and discussion

Here, we examine the influence of the number of input OCT B-scans (N) on the performance of two NNs: the U-net, and U-net with SE block. This investigation is conducted using validation datasets, encompassing both baseline (unheated) and heated datasets.

**Fig. 5.**
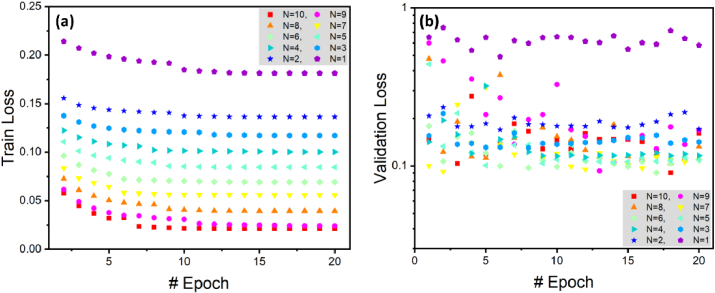
Conventional U-net performance across varied input B-scan configurations for training (a) and validation (b) datasets from epochs 1 to 20, revealing observed overfitting and convergence issues.

Figure 5 provides a comprehensive overview of the conventional U-net's performance across varying input B-scan configurations (N). This figure offers insights into specific facets of the network's behavior during training and validation.

In Fig. 5(a), the training loss exhibits a consistent decrease from epochs 1 to 20 as N increases from 1 to 10. This pattern emphasizes the capacity of U-net for improved learning with an expanded set of input B-scans, highlighting a positive correlation between the quantity of input data and the network's training performance. In Fig. 5(b), the validation loss is monitored over the same epochs with N ranging from 1 to 10. Notably, there is a pattern of high oscillation and limited loss convergence. A comparative analysis between Fig. 5(a) and Fig. 5(b) reveals substantial differences between validation and training losses for each N. These discrepancies are indicative of overfitting and raise concerns about the model's generalization capabilities.

To assess the impact of the number of input scans (N) on NN performance, employing MSE alone as a metric may be inadequate. MSE does not inherently accommodate variations in scale between predictions and ground truth labels, which may arise due to differences in N values. To address this limitation, it is useful to normalize the data before computing MSE. As discussed previously, one effective normalization strategy involves scaling both the NN predictions and ground truth labels to a common range, such as the grayscale range of [0, 255]. This normalization procedure facilitates a more informative evaluation of model performance across varying N values.

To compare NN performance later in this paper, we utilize PSNR (Eq. (1)) and SSIM (Eq. (3)). In calculating PSNR, we adopt the normalized MSE (Eq. (2)) rather than the raw MSE. This adjustment enables a more insightful comparison, revealing that increased N values generally correspond to higher PSNR and SSIM scores. This underscores the significance of employing appropriate normalization techniques for accurately assessing NN performance across diverse input configurations.

**Fig. 6.**
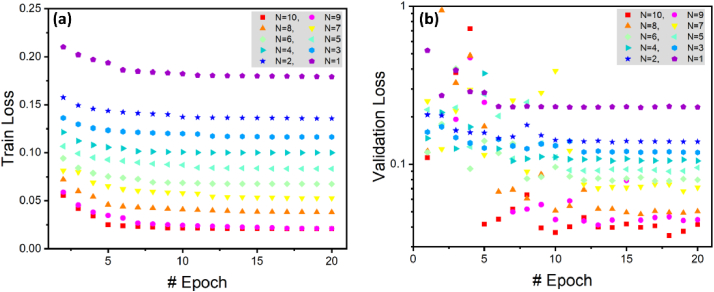
U-net with SE block performance across varied input B-scan configurations for training (a) and validation (b) datasets from epochs 1 to 20, demonstrating enhanced convergence and reduced overfitting issues.

In Fig. 6, we explore the performance of the NN incorporating the SE block. In Fig. 6(a), we examine the training loss across epochs 1 to 20 using the NN with SE block. Similar to Fig. 5(a), we observe a consistent decrease in the training loss as the number of input B-scans (N) increases. This implies that the NN with SE block benefits from a larger set of input information.

In Fig. 6(b), analogous to Fig. 5(b), we investigate the validation loss for 20 epochs using the NN with SE block. We can see that the incorporation of the SE block contributes to smoother learning, minimizing fluctuations and improving validation loss across all N values for epochs 10 and beyond. This enhancement is attributed to the SE block's ability to assign importance to each input channel, considering their relationships and relevance in predictions.

**Fig. 7.**
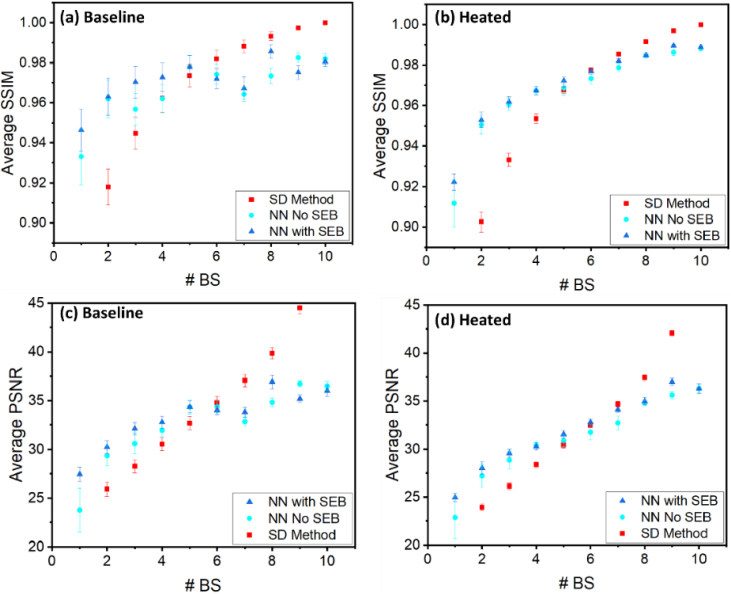
Comparing the performance of conventional Unet (light blue circle), Unet with SE block (dark blue triangle), and the speckle decorrelation (SD) algorithm (red square) using standard metrics (PSNR and SSIM) against ground truth labels. Evaluation is conducted across varying numbers of input OCT B-scans (N) from baseline and heated test datasets (Table 1), with SSIM values presented in Fig. 7(a)-(b) and PSNR values in Fig. 7(c)-(d).

In this section, we conduct a comparative analysis between Unet and Unet with SE Block, as well as the traditional speckle decorrelation algorithm. Our assessment relies on the standard metrics PSNR and SSIM, which are computed between the outputs of the three methods and the ground truth label data. The evaluation encompasses test datasets comprising varying numbers of input OCT B-scans (N), including both baseline and heated datasets (Table 1). The results are presented in Fig. 7(a)-(d), where Fig. 7(a) and 7(b) depict SSIM values for the baseline and heated datasets, respectively, and Fig. 7(c) and 7(d) illustrate PSNR values for the same datasets.

Our findings reveal a consistent improvement in PSNR and SSIM values with increasing N for the heated sample (Fig. 7(b) and 7(d)). However, for the baseline sample, particularly at higher N values, we observe fluctuations in metrics (Fig. 7(c) and 7(d)). Notably, the baseline sample demonstrates less sensitivity to changes in N compared to the heated sample, indicating a smaller slope of change in PSNR and SSIM values. This discrepancy can be attributed to the lower blood flow in the baseline sample, resulting in less pronounced changes in speckle decorrelation and subsequently less impact on the NNs’ performance with varying N.

It is noteworthy that for N values below 6, both NN architectures outperform the traditional speckle decorrelation algorithm, highlighting their ability to better utilize local information. For example, in both the baseline and heated datasets, when the conventional Unet and Unet with SE block have an input of 2 B-scans, their performance closely matches that of the speckle decorrelation algorithm with 4 B-scans. This advantage implies that fewer B-scans may be required to achieve accurate images, reducing the acquisition time during which patients must remain unmoving and consequently minimizing the risk of motion artifact due to involuntary patient movement. This advantage stems from the NNs’ inherent capacity to assimilate intricate patterns and features from the data. However, for N values equal to or greater than 6, the speckle decorrelation algorithm exhibits performance that is superior to the NNs. Several factors contribute to this phenomenon. Firstly, SNR of the estimate of speckle decorrelation improves with increasing N by a factor of $\sqrt{N}$ . Additionally, as the number of input B-scans rises, the complexity of the input data increases. This results in neural networks showing less improvement with higher N values due to their tendency to overfit specific patterns. Consequently, the slope of performance improvement with respect to N is smaller for NNs compared to the speckle decorrelation algorithm. We conjecture that utilizing larger datasets and more complex neural network architectures may have the potential to shift the crossover point to higher N values.

In the heated samples, where local heating has induced vasodilation in the skin microvasculature and an increase in blood flow, the Unet with SE block outperforms the conventional Unet. However, in the baseline sample, there are instances, especially at higher N values, where the conventional Unet performs better. This disparity can be attributed to the baseline sample's lower blood flow, which makes the SE block weighting scheme in Unet with SE block less impactful.

**Fig. 8.**
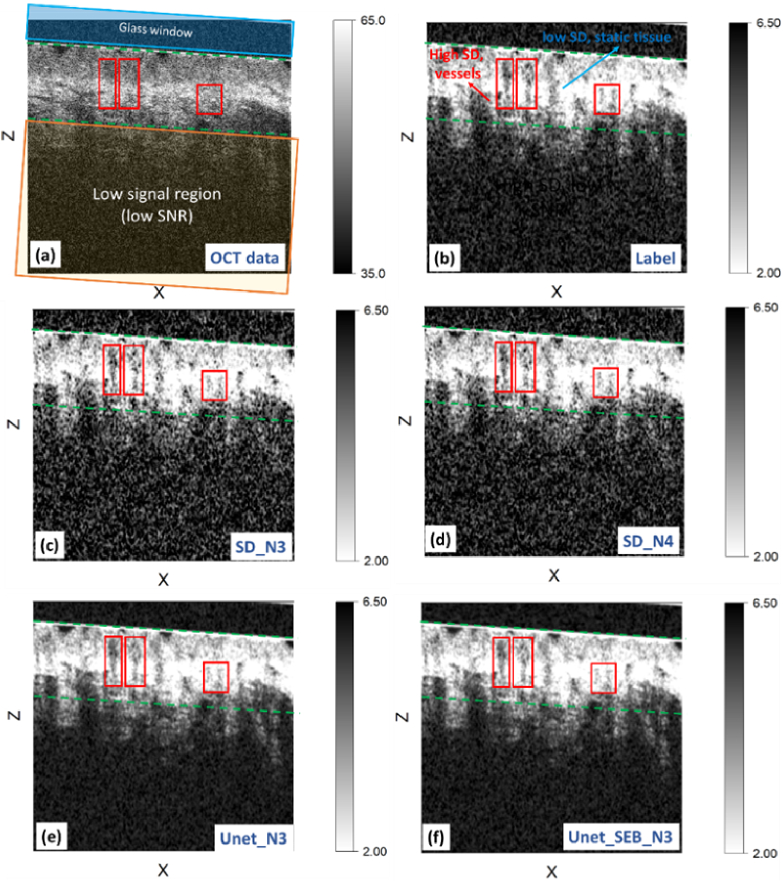
(a) The 2D cross-sectional OCT B-scan along one of the points of the slow axis. The glass window used in the experiment has been highlighted by the blue tilted rectangular. The dashed green lines denote the high SNR region of interest, while the area below marked with an orange rectangle indicates low SNR. (b) Ground truth label generated using the speckle decorrelation method. (c-f) Reconstructed images from three methods: speckle decorrelation with N = 3 (c) and N = 4 (d), conventional Unet with N = 3 (e), and Unet with SE block with N = 3 (f). Red rectangles highlight regions of high decorrelation, mainly attributed to blood vessels, within the region of interest.

In this section, we present the speckle decorrelation images generated by three distinct methods: the conventional Unet NN, the Unet with SE block, and the traditional speckle decorrelation method. Figure 8 illustrates a visual comparison of the reconstructed images. Specifically, Fig. 8(a) displays the first OCT B-scan within a series of ten consecutive B-scans obtained from the heated sample, selected at one location along the slow axis. A glass window, highlighted in the image, was placed on the skin during scanning to provide a flat tissue surface and reduce artifacts created by surface irregularities [38]. A small amount of glycerol was also placed between the glass window and the tissue to reduce mismatches in the refractive index and remove air bubbles. Note that the glass window was placed at a 4° angle from perpendicular to the light beam to reduce parasitic reflections from the air-glass and glass-glycerol interfaces. The penetration depth of optical coherence tomography varies with wavelength [39–41] and skin properties [42], most significantly impacted by the optical scattering of the tissue. Other factors include epidermal thickness and the presence of structures such as hair follicles and glands, inflammation, edema, and pigmentation levels [42,43]. Using an OCT system with a central wavelength of 1300 nm, the image penetration is approximately 1.5 mm in the skin [44]. Our experience has been that we have a sufficient signal-to-noise ratio to detect microvasculature to a depth of approximately 1 mm.

Two dashed green lines in Fig. 8(a) delineate regions representing high-signal areas of tissue in which the OCT signal has undergone minimal attenuation with depth. These regions were cropped to calculate the PSNR and SSIM metrics of the methods. Notably, regions below these delineated areas exhibit poor SNR due to attenuation of the OCT signal with optical scattering in the tissue and were excluded from the analysis. Figure 8(b) depicts the speckle decorrelation map, generated by applying the speckle decorrelation algorithm to all 10 co-located B-scans, serving as the label or ground truth for the analysis. In this map, bright regions indicate areas with low decorrelation, while dark regions represent areas with high decorrelation. The regions above and below the selected area between the dashed lines exhibit high decorrelation (dark region in Fig. 8) due to low SNR values. Within the area located between the dashed green lines, we observe a combination of bright regions representing static tissues and dark regions indicating high decorrelation, primarily attributed to blood flow. Several regions exhibiting high decorrelation indicative of blood vessels are delineated by red rectangles. Figure 8(c), 8(d), 8(e), and 8f illustrate the generated images obtained through various processes. Figure 8(c) shows the image generated by applying the speckle decorrelation algorithm to three B-scans as input (labeled as SD_N3). In Fig. 8(d), the image results from applying the speckle decorrelation algorithm to four B-scans (labeled as SD_N4). Figure 8(e) displays the output produced by the conventional Unet, while Fig. 8(f) presents the result generated by the Unet with SE block, both using three B-scans as input (labeled as Unet_N3 and Unet_SE_N3, respectively).

**Fig. 9.**
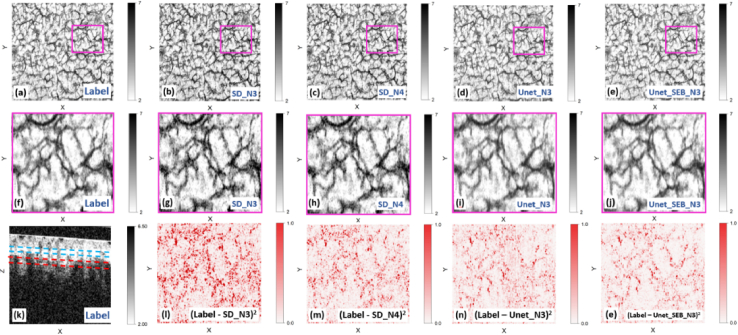
Comparative analysis of blood vessel predictions utilizing Maximum Intensity Projection (MIP) images and squared error assessment. The first row presents MIP images alongside the label (a) and predictions from SD_N3 (b), SD_N4 (c), Unet_N3 (d), and Unet_SE_N3 (e) methods, calculated between the blue dashed lines shown in Fig. 9(k). The second row shows magnified views of the regions inside the pink rectangles in the first row (Fig. 9(f)-(j)). Squared error analysis between the magnified label (Fig. 9(f)) and the predictions’ magnified views (Fig. 9(g)-(j)) in the third row (Fig. 9(l)-(o)) highlights the superior performance of Neural Network (NN) methods, particularly Unet_SE_N3, in accurately delineating blood vessels compared to speckle decorrelation algorithms.

**Fig. 10.**
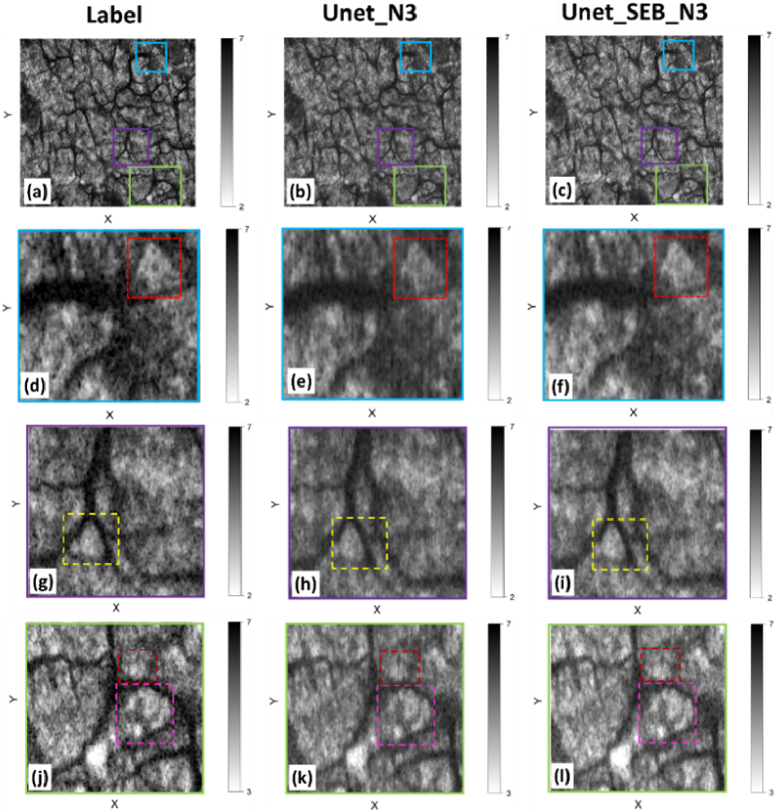
Comparative evaluation of neural networks’ performances in blood vessel detection through Maximum Intensity Projection (MIP) imaging. The top row illustrates MIP images alongside ground truth labeling (a) and predictions generated by Unet_N3 (b) and Unet_SEB_N3 (c) methodologies, analyzed within the tissue depths delineated by the red dashed lines in Fig. 9(k). Subsequent rows provide magnified views of regions demarcated by blue, purple, and green rectangles in the first row, allowing detailed scrutiny of prediction accuracies. Findings indicate that Unet_SEB_N3 demonstrates closer alignment with ground truth labeling, particularly visible in regions of low decorrelation (white areas), suggesting its potential superiority in capturing intricate blood vessel patterns.

Figure 9 shows an enface (X × Y) representation of the speckle decorrelation values, providing a clearer representation of the microvascular network. The first row of Fig. 9 displays the application of Maximum Intensity Projection (MIP) on the label image (Fig. 9(a)) alongside predictions generated by various methods: SD_N3 (Fig. 9(b)), SD_N4 (Fig. 9(c)), Unet_N3 (Fig. 9(d)), and Unet_SE_N3 (Fig. 9(e)). To examine the impact of noise, we calculated the MIP at different depths. As we penetrate deeper into the tissue, the noise level increases, presenting varying challenges for our models. We aim to observe how well the models perform under different noise levels. The MIP highlighted in Fig. 9 focuses on the region of interest within the B-scan, ranging from 60 to 90 pixels from the surface, marked by blue dashed lines in Fig. 9 k. Similarly, in Fig. 10, the MIP targets the region spanning from 120 to 150 pixels from the surface, indicated by red dashed lines.

Comparing the images shows that the CNN methods, Unet_N3 and Unet_SE_N3, demonstrate superior SNR and offer clearer delineation of the vascular network. To comprehensively evaluate method performance, squared error analysis was conducted between the label (Fig. 9(f)) and corresponding predictions by SD_N3 (Fig. 9(g)), SD_N4 (Fig. 9(h)), Unet_N3 (Fig. 9(i)), and Unet_SEB_N3 (Fig. 9(j)), with results presented in the third row of Fig. 9. Red regions denote areas of high error, indicating discrepancies between predictions and the label, while white regions represent accurate predictions in alignment with the label.

Interestingly, NN predictions demonstrate fewer error regions (red) compared to speckle decorrelation algorithms using comparable numbers of B-scans (N = 3 and N = 4). Despite utilizing four input B-scans in SD_N4, it still exhibits more red regions than both NN predictions, emphasizing the NNs’ superior performance.

In Fig. 10, we expanded our analysis to explore the performance of the two NN models at different depths within the tissue, aiming to gain deeper insights into their ability to accurately identify blood vessels under varying noise levels. Specifically, the MIP was calculated for the region spanning from 120 to 150 pixels from the surface, as indicated by the red dashed lines in Fig. 9(k). The first row of Fig. 10 presents three images depicting the MIP for the label (Fig. 10(a)), Unet_N3 (Fig. 10(b)), and Unet_SEB_N3 (Fig. 10(c)), respectively. To provide a closer examination of the model predictions, we selected three specific regions marked by blue, green, and purple rectangles within each full-scale image. Magnified (zoomed) images of these regions are presented in the three rows below each full-scale image. In the depicted images, dark areas indicate regions with pronounced decorrelation, suggesting the presence of blood vessels. Conversely, light regions denote areas with low decorrelation, implying temporally invariant speckle intensity patterns, indicating stationary (non-vascular) tissue. The presence of darker regions amidst vessels suggests a lower SNR, underscoring the impact of noise on the imaging process. These darker regions, stemming from speckle alterations induced by noise, pose challenges for precise blood vessel detection. Interestingly, as we get deeper into the tissue layers, the prevalence of these dark regions due to low SNR seems to increase. This observation hints at the increasing impact of multiple optical scattering in deeper tissue regions, potentially impeding the models’ ability to precisely identify blood vessels. The images reveal that the NNs have contributed to smoothing the images, evident in the more uniform intensities of the vessels and the higher regions between vessels compared to the label image. However, upon comparing the results of Unet_SEB_N3 and Unet_N3, we observe that the values predicted by Unet_SEB_N3 are closer to the label, with less pronounced artificial smoothing effects. For instance, in each of the three regions highlighted by dashed rectangular boxes, predominantly located in low decorrelation values in the label image (white areas), we can see that the regions predicted by Unet_SEB_N3 are closer to the label and display fewer dark points. This observation indicates the superiority of Unet_SEB_N3 over the conventional Unet_N3 to reflect the true decorrelation values more accurately.

To provide a more comprehensive assessment and comparison of method performance, we computed the MSE between the MIP image of the label and the MIP image generated by each method's prediction. These results are detailed in Table 2. Furthermore, Table 2 includes the evaluation metrics of PSNR and SSIM, offering additional insights into the quality and fidelity of the methods’ outputs compared to the ground truth label.

**Table 2.** Comparison of Method Performance Using MSE, PSNR, and SSIM Metrics

Metric	SD_3N	SD_4N	Unet_3N	Unet_SE_3N
MSE of the MIP images (Fig. 9)	0.2819	0.1701	0.1252	**0** **.** **1183**
PSNR at the selected slow axis point (Fig. 8)	29.08	31.23	32.91	**33**.**18**
SSIM at the selected slow axis point (Fig. 8)	0.9378	0.9581	0.9652	**0**.**9661**
Average PSNR in the heated test dataset	29.10	31.24	32.59	**33**.**10**
Average SSIM in the heated test dataset	0.9378	0.9577	0.9629	**0**.**9642**

Both the NN methods consistently outperformed the traditional speckle decorrelation method, even when a smaller number of input B-scans were used. This observation is highlighted by lower MSE values and higher PSNR and SSIM scores achieved by the NN-based approaches. A comparative analysis between the two NN methods reveals that employing the SE block has enhanced the performance of the NN models.

## 5. Conclusion

In this study, we introduced a novel approach for detecting and analyzing skin microvasculature using deep learning-enhanced optical coherence tomography angiography (OCTA) reconstruction. By integrating a CNN with a SE block, our method efficiently leverages local information to enhance accuracy and reduce measurement time, overcoming challenges posed by traditional methods that often encounter motion artifacts.

Our findings demonstrate the superiority of neural networks over traditional speckle decorrelation algorithms, particularly in scenarios with a low number of co-located B-scans. By capitalizing on spatial relationships between pixels and the inherent continuity of microvessel structures, our approach surpasses methods heavily reliant on sliding filters.

Furthermore, the integration of the SE block not only improves stability and reduces overfitting but also enhances accuracy compared to conventional CNN architectures. The SE block dynamically recalibrates features, allowing the network to adaptively assign varying importance to different channels based on their relevance, better capturing the relationships between B-scans acquired at the same location.

Our analysis of the proposed method explores its performance at different tissue depths and noise levels. Our findings underscore the superior accuracy of incorporating the SE block with a Unet, particularly in identifying blood vessels amidst noise-induced challenges and mitigating artificial smoothing effects. A limitation of this study is that our evaluation has focused on the use of OCT to assess skin microvasculature. Other clinical applications for these algorithms include retinal imaging, which has significantly different requirements in terms of image acquisition speed and the optical properties of the surrounding tissue.

Overall, our study contributes to advancing microvascular imaging by introducing a deep learning-based approach for OCTA reconstruction. The efficiency, accuracy, and stability of our method hold promise for improving the detection and analysis of skin microvasculature, with potential applications in dermatologic and systemic health.

## Data Availability

The data that support the findings of this study are available from the corresponding author upon reasonable request.
